# Associations between serum 25(OH)D concentrations and prevalent asthma among children living in communities with differing levels of urbanization: a cross-sectional study

**DOI:** 10.1186/s40733-017-0033-2

**Published:** 2017-06-02

**Authors:** Suzanne L. Pollard, John J. Lima, Karina Romero, Carla Tarazona-Meza, Edward Mougey, Katherine Tomaino, Gary Malpartida-Guzmán, Nadia N. Hansel, William Checkley, GASP Study Investigators, Patrick Breysse, D’Ann Williams, Caroline Johnson, Sonali Bose, Lindsay Underhill, Rocío Galvez, Chen Chen

**Affiliations:** 1 Division of Pulmonary and Critical Care, School of Medicine, Johns Hopkins University, 1800 Orleans Ave, Suite 9121, Baltimore, USA; 2 Department of International Health, Bloomberg School of Public Health, Johns Hopkins University, Baltimore, USA; 3 Center for Pharmacogenomics and Translational Research, Nemours Children’s Health System, Jacksonville, FL USA; 4 Biomedical Research Unit, A.B. PRISMA, Lima, Peru

**Keywords:** Asthma, Pediatric asthma, Vitamin D, 25(OH)D, Nutrition, Urbanization

## Abstract

**Background:**

Prior evidence suggests that vitamin D deficiency may increase the risk of asthma and atopy and impair pulmonary function in children.

**Methods:**

In this cross-sectional analysis nested in a case-control study, we analyzed serum 25(OH)D concentrations in 413 children with asthma and 471 children without asthma living in two geographically adjacent study communities (Pampas and Villa El Salvador). We measured total and antigen-specific IgE levels, pulmonary function, asthma control, and exhaled nitric oxide.

**Results:**

Mean 25(OH)D concentrations were 25.2 ng/mL (SD 10.1) in children with asthma and 26.1 ng/mL (SD 13.7) in children without asthma (*p* = 0.28). Vitamin D deficiency (25(OH)D < 20 ng/ml) was more common in Pampas than in Villa El Salvador (52.7% vs. 10.5%; *p* < 0.001). In the overall study population, a 10 ng/ml decrease in serum 25(OH)D concentrations was not significantly associated with odds of asthma (OR 1.09, 95% CI: 0.94 to 1.25). However, vitamin D deficiency was associated with a 1.6-fold increase in odds of asthma in the overall cohort (95% CI: 1.14 to 2.25). After stratifying by site, a 10 ng/mL decrease in serum 25(OH)D concentrations was associated with 18% higher odds of having asthma in Pampas (OR = 1.18, 95% CI 1.02 to 1.38), whereas there was no significant association between 25(OH)D concentrations and asthma in Villa El Salvador (OR = 0.95, 95% CI 0.87 to 1.05). Combined data from these geographically adjacent populations suggests a possible threshold for the relationship between 25(OH)D levels and asthma at approximately 27.5 ng/ml. Serum 25(OH)D concentrations were not clearly associated with asthma control, total serum IgE, atopy, or airway inflammation.

**Conclusion:**

Serum 25(OH)D concentrations were inversely associated with asthma in one study community with a high prevalence of deficiency. Studies are needed to investigate a possible threshold 25(OH)D concentration after which higher vitamin D levels show no further benefit for asthma.

**Electronic supplementary material:**

The online version of this article (doi:10.1186/s40733-017-0033-2) contains supplementary material, which is available to authorized users.

## Background

Asthma is a chronic lung disease characterized by airway inflammation, airflow limitation, bronchial hyper-responsiveness, and episodic wheeze and cough. An estimated 334 million individuals worldwide are currently living with asthma [1]. Asthma is the most common chronic disease in childhood, with an estimated 14% of children worldwide experiencing asthma symptoms in the previous year [1]. Recent research has focused on understanding the relationship between vitamin D deficiency and asthma and the potential benefits of vitamin D supplementation in improving a variety of asthma-related outcomes.

Vitamin D may play a role in modulating risk and severity of asthma through its functions as an immune modulator, its role in lung development, and the effects of vitamin D deficiency on lung impairment and airway remodeling [2, 3]. Vitamin D may also influence epigenetic programming in early life and may influence asthma severity and control through its anti-inflammatory properties and its potential role in reversing steroid resistance [4, 5]. However, epidemiological studies on the relationship between vitamin D status and asthma, including cross-sectional and prospective studies, and randomized supplementation trials [6], have been conflicted.

Given the relative lack of large-scale studies of asthma and vitamin D in low- and middle-income countries, we sought to examine the relationship between vitamin D deficiency and prevalent asthma in a cohort of children and adolescents living in two peri-urban communities of Lima, Peru. An earlier study by our research group found an independent relationship between total serum 25(OH)D levels and asthma prevalence in children [7]. The current analysis was ancillary to a parent study of the role of genetic factors in modifying the relationship between air pollution and asthma. For the current analysis, we measured 25(OH)D levels in 413 children with asthma and 471 healthy controls, and we hypothesized that serum 25(OH)D concentrations would be inversely associated with odds of asthma, asthma severity, and markers of airway inflammation and allergy, and positively associated with pulmonary function.

## Methods

### Study population and setting

The study population was drawn from two communities, Pampas de San Juan de Miraflores (Pampas) and Villa El Salvador (Villa), located approximately 25 km south of the city center of Lima, Peru. Pampas and Villa have grown rapidly over the last two decades. As compared to Villa, a higher proportion of inhabitants in Pampas were born in the highlands, and a lower proportion were native to Lima. The two communities differ in age structure and socioeconomic status (SES), with Pampas being less urbanized, having a younger population, and having lower SES overall. However, the main economic activities between these two communities are similar. We chose to carry out this study in two communities to increase our recruitment pool of asthma cases. Annual average precipitation in Lima is less than 50 mm per year, and the city has high cloud cover 9 months of the year. A study conducted in 2010 by our group in 725 adolescents 13–15 years of age living in Pampas found that 22% of participants had lifetime wheeze, 12% had asthma symptoms, and 13% had a physician diagnosis of asthma [8]. This study was approved by the Institutional Review Boards at Johns Hopkins University School of Medicine, Baltimore, USA, and A.B. PRISMA in Lima, Peru.

### Study design

This is an ancillary analysis of a subset of children enrolled in an unmatched case-control study carried out between July 2012 and March 2014. We enrolled children aged 9 to 19 years living in the two study communities. We excluded children with ocular, abdominal, or thoracic surgery in the past 3 months, hospitalization for cardiac reasons in the past 3 months, diagnosis or current treatment for tuberculosis, a chronic respiratory condition other than asthma, or were pregnant at enrollment. We recruited participants using household community census surveys. We identified and visited all potential asthma cases aged 9 to 19 years in the two communities using birthdate and a positive response to a census question identifying individuals with wheeze or use of asthma medications in the past 12 months, or a lifetime physician diagnosis of asthma. We identified children without asthma using a simple random sample of children aged 9 to 19 years in our census that responded negatively to all asthma-related census questions. We confirmed asthma status at enrollment and evaluated asthma severity in accordance with NAEPP-3 guidelines [9]. For this ancillary analysis, we defined children with asthma as having self- or parental-report of any occurrence of wheezing in the chest or any use of asthma medications in the past year. We defined children without asthma as no occurrence of self- or parentally-reported wheeze symptoms consistent with asthma in the past year and no use of asthma medications in the past year. Children with previous asthma were defined as children who had a physician diagnosis of asthma but had no symptoms or medication use related to asthma in the previous year; children with previous asthma were included as part of the non-asthma group for primary analyses. Per these definitions, we had a final enrollment of 258 and 248 children with asthma, and 374 and 297 without asthma, in Pampas and Villa, respectively.

### Questionnaires

We administered a baseline questionnaire, which included questions regarding demographic information, socioeconomic status, asthma medication use, history of allergic rhinitis and eczema, and smoking history.

### Clinical measurements

We conducted spirometry at enrollment in all participants. We used a flow-based portable spirometer (SpiroPro, Jaeger/ERT, Hoechberg, Germany), obtaining at least three acceptable and reproducible spirometry maneuvers for a maximum of eight according to ATS/ERS guidelines [10]. We calculated predicted values and Z-scores using multi-ethnic reference values derived by the Global Lung Health Initiative [11]. We measured Asthma Control Test (ACT) score using a validated questionnaire [12–14]. An ACT score ≥ 20 is indicates controlled asthma, 16–19 is considered partially controlled, and <16 is considered uncontrolled [14]. We measured fractional exhaled nitric oxide (FeNO) using the handheld NIOXMINO (Aerocrine, Solna, Sweden). We used criteria derived by the World Health Organization to determine Body Mass Index (BMI)-for-age Z-scores [15].

### Measurement of serum vitamin D levels

We collected one blood sample per participant using standard phlebotomy techniques. We separated and centrifuged samples within two hours of extraction and stored at −80 °C. We quantified serum 25(OH)D levels at the Analytical Laboratory at Nemours Children Health System, Jacksonville, FL using the ALPCO 25OH Vitamin D Total ELISA kit (ALPCO, Salem, NH). Body composition measures were determined through bioimpedance using the TANITA TBF-300 body composition analyzer (TANITA Corporation, Inc., Arlington Heights, IL). We measured both total serum Immunoglobulin E (IgE) and IgE specific antibodies to mixes of three common allergens (animal, mold, and dust mite) using the ImmunoCAP 250 (ThermoFisher Scientific, Kalamazoo, MI). An IgE level of > 0.1 kU/L indicated a positive IgE antibody response, and a positive response to any of the three mixes indicated atopy.

### Definition of vitamin D status

We defined vitamin D deficiency as total serum 25(OH)D levels below 20 ng/ml, and vitamin D insufficiency as a level between 20 and 30 ng/ml [16]. Atopy was defined as the presence of IgE antibody to mixes of one or more of three common allergens (mold, dust mite, and animal).

### Biostatistical methods

We sought to determine associations between 25(OH)D serum concentrations and prevalent asthma and to determine associations between pulmonary function, FeNO, and allergy with 25(OH)D levels. For the parent study, our goal was to enroll 700 asthma cases and 700 controls. With this sample size, we could detect an odds ratio of 1.6 with a disease allele of frequency 0.1 with 80% power. We used multivariable logistic regressions to model the association between asthma and 25(OH)D and vitamin D deficiency after adjusting for season of blood draw, age, sex, BMI, and SES score, and conducted both combined analyses and stratified by atopy. We used multivariable linear regressions to model associations between total 25(OH)D levels and pre-bronchodilator forced expiratory volume in 1 second (FEV_1_), forced vital capacity (FVC), FeNO, total serum IgE, and ACT score. We used the chi-square test to compare proportions between children with and without asthma, and analysis of variance to compare means. We used a smoothing spline to visualize the relationship between unadjusted odds of asthma and serum 25(OH)D concentrations. We conducted a sensitivity analysis to determine if removing children with previous asthma (i.e. children with a previous asthma diagnosis but no current symptoms or medication use) affected the association between serum 25(OH)D concentrations and asthma (Additional file 1: Table S1). We generated a composite score for SES using Principal Component Analysis techniques. We used random forest methods to impute missing observations for SES (<10% of data). For all other analyses, participants with missing data were omitted. We conducted analyses in R (http://www.r-project.org) and STATA 11 (Stata Corp., College Station, Texas).

## Results

### Characteristics of the study population

Of 1177 children enrolled, 884 (413 with asthma, 471 without asthma) had serum available for analysis of 25(OH)D levels. There were no differences in age (13.4 vs. 13.5 years, *p* = 0.48), sex (50.9% vs. 53.4% male, *p* = 0.45), atopy (69.7% vs. 79.0% with atopy, *p* = 0.39), pre-bronchodilator FEV_1_ Z-score (1.3 vs. 1.4, *p* = 0.57), or body mass index (21.9 vs. 21.5, *p* = 0.13) between individuals with and without a blood sample, respectively. There was a significant difference in the proportion with asthma (46.7% vs. 31.7%, *p* < 0.001) in children with and without a blood sample. Among the 884 children, mean age at enrollment was 13.4 years (SD = 2.65), 53.4% were boys, and 52.6% lived in Pampas. There were differences in SES indicators between study communities, but the proportion of children with asthma was similar.

We summarized baseline characteristics of children with asthma as compared to controls (Table 1). Children with asthma had higher BMI and BMI-for-age Z-score. They also differed by indicators of SES and calendar quarter of blood draw. Asthma was associated with a lower pre-bronchodilator FEV_1_ Z-score and FEV_1_/FVC Z-score, but not with pre-bronchodilator FVC Z-score. Children with asthma had a higher prevalence of atopy, self-reported allergy and eczema, parental history of asthma and allergic rhinitis, and FeNO levels than controls. Mean total 25(OH)D concentrations were 25.7 ng/ml in all children enrolled in our study. Mean 25(OH)D concentration did not differ between participants with and without asthma (Table 1).

**Table 1:** Participant characteristics among children with and without asthma

	Children with Asthma	Children without asthma	*p*-value
Sample size	413	471	
Lives in Pampas de San Juan, n (%)	374 (55.7)	258 (51.0)	0.11
Vitamin D Measures			
Total 25(OH)D (ng/ml), mean (SD)	25.2 (10.1)	26.1 (13.7)	0.28
Demographics			
Age in years, mean (SD)	13.9 (2.65)	14.0 (2.77)	0.43
n, (%) boys	234 (56.7)	238 (50.5)	0.07
Anthropometry			
Height in cm, mean (SD)	150.9 (12.0)	151.0 (12.0)	0.99
BMI in kg/m^2^, mean (SD)	22.2 (4.23)	21.7 (4.07)	0.03
BMI-for-age z-score, mean (SD)	1.03 (1.2)	0.83 (1.1)	0.01
Socioeconomics, n (%)			
Maternal education ≥ 6 years	274 (71.0)	269 (60.5)	0.001
6 or more household members	200 (51.6)	232 (51.6)	0.99
SES Score	0.243 (1.68)	−0.183 (1.66)	<0.001
Smoking			
Current smoker	6 (1.8)	10 (2.4)	0.56
Pulmonary function, allergy, FeNO			
Pre-BD FEV_1_ z-score, mean (SD)	1.08 (1.40)	1.55 (1.16)	<0.001
Pre-BD FVC z-score, mean (SD)	1.48 (1.41)	1.34 (1.21)	0.69
Pre-BD FEV_1_/FVC z-score, mean (SD)	−0.355 (−1.16)	0.413 (0.837)	<0.001
Either parent with history of physician-diagnosed asthma, n (%)	98 (25.7)	48 (11.1)	<0.001
Atopy, n (%)	488 (62.3)	315 (78.2)	<0.001
Pollen allergy (self-report), n (%)	38 (9.79)	11 (2.51)	<0.001
Animal dander allergy (self-report), n (%)	107 (27.6)	30 (6.73)	<0.001
Ever having allergic rhinitis (self-report), n (%)	291 (75.2)	171 (38.0)	<0.001
Either parent with history of allergic rhinitis (self-report), n (%)	124 (32.6)	88 (20.2)	<0.001
Ever having eczema (self-report), n (%)	53 (13.7)	28 (6.25)	<0.001
Either parent with history of eczema (self-report), n (%)	12 (3.15)	13 (3.00)	0.90
Fractional exhaled nitric oxide in ppb, mean(SD)	34.3 (32.9)	20.0 (21.4)	<0.001
Calendar quarter at time of blood draw, n, (%)			
January–March	102 (24.7)	89 (18.9)	<0.001
April–June	13 (3.15)	9 (1.91)	
July–September	73 (17.7)	4 (0.85)	
October–December	225 (54.5)	369 (78.3)	
Mean daily frequency of vitamin D-rich food intake in 14 days prior to blood draw, mean (SD), *n* = 646			
Vitamin-D rich oily fish	0.13 (0.15)	0.13 (0.16)	0.89
Vitamin-D fortified milk	0.55 (0.54)	0.54 (0.62)	0.77

We summarized characteristics of children living in Pampas and Villa (Table 2). Children living in Pampas had significantly lower 25(OH)D concentrations and were slightly older. Children living in Villa had a significantly higher percentage of mothers with 6 or more years of education, as well as a higher composite SES score. Calendar quarter of blood draw differed between the two sites.

**Table 2:** Comparison of participant characteristics among children by study community

	Pampas	Villa	*p*-value
Sample size	419	465	
Asthma status, n (%)	258 (40.8)	248 (45.5)	0.11
Vitamin D Measures			
Total 25(OH)D (ng/ml), mean (SD)	20.9 (13.1)	30.0 (9.4)	<0.001
Demographics			
Age in years, mean (SD)	14.5 (2.69)	13.4 (2.62)	<0.001
n, (%) boys	341 (54.0)	280 (51.4)	0.21
Anthropometry			
Height in cm, mean (SD)	151 (12.2)	151 (11.8)	0.74
BMI in kg/m^2^, mean (SD)	21.8 (3.75)	22.1 (4.48)	0.34
BMI-for-age z-score, mean (SD)	0.840 (1.1)	1.00 (1.22)	0.04
Socioeconomics, n (%)			
Maternal education ≥ 6 years	210 (52.9)	333 (76.7)	<0.001
6 or more household members	191 (47.8)	241 (55.0)	0.04
SES Score	−0.684 (1.57)	0.793 (1.45)	<0.001
Smoking			
Current smoker	11 (3.06)	5 (1.23)	0.08
Pulmonary function, allergy, FeNO			
Pre-BD FEV_1_ z-score, mean (SD)	1.36 (1.27)	1.31 (1.31)	0.57
Pre-BD FVC z-score, mean (SD)	1.44 (1.26)	1.28 (1.34)	0.07
Pre-BD FEV_1_/FVC z-score, mean (SD)	−0.0296 (1.09)	0.129 (1.06)	0.03
Either parent with history of physician-diagnosed asthma, n (%)	69 (17.9)	77 (18.0)	0.95
Atopy, n (%)	293 (71.3)	310 (68.3)	0.34
Pollen allergy (self-report), n (%)	27 (6.94)	22 (5.02)	0.24
Animal dander allergy (self-report), n (%)	71 (17.9)	66 (15.1)	0.27
Ever having allergic rhinitis (self-report), n (%)	229 (57.5)	233 (53.2)	0.21
Either parent with history of allergic rhinitis (self-report), n (%)	98 (25.3)	114 (26.6)	0.68
Ever having eczema (self-report), n (%)	56 (14.1)	25 (5.71)	<0.001
Either parent with history of eczema (self-report), n (%)	18 (4.63)	7 (1.64)	0.01
Exhaled nitric oxide in ppb, mean(SD)	29.6 (30.8)	23.2 (24.6)	0.001
Calendar quarter at time of blood draw, n, (%)			
January–March	0	191 (41.1)	<0.001
April–June	0	22 (4.73)	
July–September	77 (18.4)	0	
October–December	342 (81.6)	252 (54.2)	
Mean daily frequency of vitamin D-rich food intake in 14 days prior to blood draw, mean (SD); *n* = 646			
Vitamin-D rich oily fish	0.12 (0.16)	0.14 (0.16)	0.04
Vitamin-D fortified milk	0.51 (0.46)	0.59 (0.66)	0.04

### Distributions of 25(OH)D concentrations, BMI-for-age Z-score, and SES score by study community

**Fig. 1:**
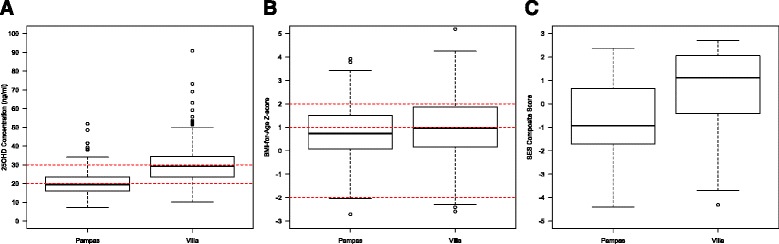
Distributions of total 25(OH)D concentrations (**a**), BMI-for-Age Z-scores (**b**), SES Composite Score (**c**) between communities. *Red horizontal dashed lines* in 1a represent concentrations for vitamin D deficiency and insufficiency; *dashed lines* in 1b represent cutoffs for underweight (−2), overweight (+1), and obese (+2)

A higher proportion of the study population was vitamin D deficient in Pampas as compared to Villa (52.7% vs. 10.5%, *p* < 0.001) (Fig. 1a). A lower proportion of individuals in Pampas as compared to Villa had BMI-for-age Z-scores in the overweight or obese categories (39.1% vs. 48.9%, *p* = 0.01) (Fig. 1b), and SES composite score was lower overall in Pampas vs. Villa (−0.68 vs. 0.79, *p* < 0.001), indicating significantly higher SES in Villa (Fig. 1c).

### Relationship between total serum 25(OH)D concentrations and asthma

**Fig. 2:**
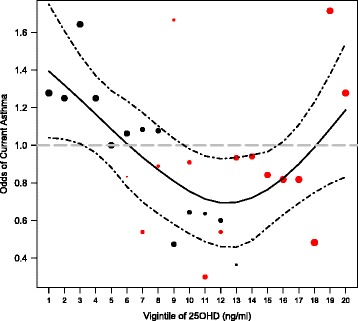
Unadjusted odds of asthma for each vigintile of 25(OH)D concentrations. The *solid line* represents the lowess smoothed curve and the *dashed lines* represent confidence intervals for this curve. The *black dots* indicate the odds of asthma in Pampas de San Juan, and the *red dots* indicate the odds of asthma in Villa El Salvador. The size of the dot is proportional to the number of individuals included in calculation of the odds. Between vigintiles 1 through 13 (approximately 27.5 ng/ml), we observed an inverse relationship between odds of asthma and 25(OH)D levels. Between vigintiles 13 and 20, we observed a positive relationship between odds of asthma and 25(OH)D levels

We observed a U- or L-shaped association between unadjusted odds of asthma across 25(OH)D serum levels (Fig. 2). We observed an inverse relationship between odds of asthma and 25(OH)D levels at levels below approximately 27.5 ng/ml, and we observed a positive relationship between odds of asthma and 25(OH)D levels above 27.5 ng/ml. In the overall study population, a 10 ng/ml decrease in serum 25(OH)D concentrations was not significantly associated with odds of asthma (OR 1.09, 95% CI: 0.94 to 1.25), after adjusting for season of blood draw, age, sex, BMI, atopy, site, and SES. However, vitamin D deficiency (25(OH)D < 20 ng/ml) was associated with a 1.6-fold increase in odds of asthma in the overall cohort (95% CI: 1.14 to 2.25). Stratification showed important differences in this relationship by site. Specifically, in multivariable logistic regression, a 10 ng/ml decrease in total serum 25(OH)D levels in children living in Pampas was associated with a significant 18% increase in odds of asthma when compared against healthy controls, after adjusting for season of blood draw, age, sex, BMI, atopy, and SES (Table 3). This relationship was no longer significant after stratifying by atopy, although it approached significance in children with atopy. Decreased serum 25(OH)D levels were associated with a non-significant decrease in odds of asthma in Villa. Similarly, vitamin D deficiency was associated with a 2.2-fold increase in odds of asthma in Pampas (95% CI: 1.42 to 3.28, *p* < 0.001), whereas deficiency was associated a non-significant decrease in odds of asthma in Villa (OR 0.94, 95% CI: 0.50 to 1.78, *p* = 0.86). In sensitivity analyses, removing children with previous asthma did not affect the direction of the associations or statistical significance (see Additional file 1: Table S1). However, the magnitude of the association increased upon removing previous cases in all but one association.

**Table 3:** Multivariable logistic regression analysis of the association between 25-OH vitamin D levels and asthma among 884 children and adolescents in Lima, Peru

	Adjusted^a^ ORs (95% CI) with total serum 25(OH)D levels (per 10 ng/ml decrease)		
	Overall (*n* = 884)	Atopy (*n* = 603)	No Atopy (*n* = 262)
Pampas de San Juan	1.18 (1.02 to 1.38)	1.18 (0.985 to 1.42)	1.07 (0.806 to 1.43)
	p= 0.03	p= 0.07	p= 0.63
Villa El Salvador	0.954 (0.867 to 1.05)	0.925 (0.829 to 1.03)	1.06 (0.86 to 1.32)
	p= 0.33	p= 0.16	p= 0.59

^a^Adjusted for season, age, sex, BMI, and SES. Overall model also adjusted for atopy

### Total serum 25(OH)D concentrations and pulmonary function

Pre-bronchodilator FEV_1_ Z-scores did not differ between Pampas and Villa. However, pre-bronchodilator FVC Z-score was higher in Pampas when compared to Villa (1.4 vs. 1.3 SD, *p* = 0.03). Pre-bronchodilator FEV_1_ and FVC Z-scores did not differ significantly between children with and without vitamin D deficiency. In adjusted analyses, pre-FEV_1_ Z-score and pre-FVC Z-score were not significantly associated with 25(OH)D levels overall, or after stratifying by asthma status (Table 4).

**Table 4:** Multivariable linear regression analysis of the association between 25(OH)D concentrations and measures of pulmonary function, asthma biomarkers, and asthma control

	Overall (*n* = 884)		Asthma (*n* = 413)		Healthy controls (*n* = 471)	
	Unadjusted Beta (95% CI)	Adjusted^a^ Beta (95% CI)	Unadjusted Beta (95% CI)	Adjusted Beta (95% CI)	Unadjusted Beta (95% CI)	Adjusted Beta (95% CI)
Pre-bronchodilator FEV_1_ z-score	−.129 (−0.750 to 0.493)	.0566 (−0.560 to 0.673)	0.0298 (−0.672 to 0.731)	0.153 (−0.464 to 0.769)	−0.484 (−1.56 to 0.592)	−0.178 (−1.28 to 0.92)
	p= 0.69	p= 0.86	p= 0.93	p= 0.63	p= 0.38	p= 0.75
Pre-bronchodilator FVC z-score	−0.476 (−1.09 to 0.14)	−0.256 (−0.868 to 0.356)	−0.244 (−0.940 to 0.452)	−0.155 (−0.782 to 0.472)	−0.741 (−1.77 to 0.284)	−0.455 (−1.52 to 0.607)
	p= 0.13	p= 0.41	p= 0.49	p= 0.63	p= 0.16	p= 0.40
eNO (ppb)	−.0306 (−.0610 to −0.000369)	−0.0295 (−0.0621 to 0.00308)	−0.0142 (−0.0440 to 0.0156)	−0.0.00798 (−0.0359 to 0.00199)	−0.0518 (−0.115 to 0.0113)	−0.0711 (−0.139 to 0.0305)
	p= 0.05	p= 0.08	p= 0.35	p= 0.57	p= 0.11	p= 0.04
Total serum IgE (kUa/L, log scale)	0.269 (−0.278 to 0.816)	0.0494 (−0.676 to 0.775)	0.305 (−0.392 to 1.00)	−0.229 (−1.08 to 0.618)	0.401 (−0.466 to 1.27)	0.202 (−0.926 to 1.33)
	p= 0.33	p= 0.89	p= 0.39	p= 0.60	p= 0.36	p= 0.73
ACT score	NA	NA	−0.1477 (−0.420 to 0.126)	−0.116 (−0.322 to 0.893)	NA	NA
			p= 0.29	p= 0.27		

^a^Adjusted for season, age, sex, BMI, and SES. Overall model also adjusted for asthma status

### Total serum 25(OH)D concentrations and markers of inflammation, allergy, and asthma control

In adjusted analyses, ACT score was not significantly associated with 25(OH)D levels. FeNO was significantly inversely associated with 25(OH)D levels in single variable analyses (−0.03 ng/ml, 95% CI −0.06 to −0.0004), but was not significantly associated in multivariable analyses (−0.03 ng/ml, 95% CI −0.06 to 0.003). Finally, total serum IgE was not significantly associated with 25(OH)D levels in either unadjusted or adjusted analyses (Table 4).

## Discussion

In this study of 884 Peruvian children, we found that total serum 25(OH)D levels were not associated with odds of asthma in the combined study population, while vitamin D deficiency was independently associated with odds of asthma. However, stratification by study community revealed important differences. In Pampas, a decrease in 25(OH)D levels was significantly associated with prevalence of asthma. In Villa, we observed that increased 25(OH)D serum levels were associated with a non-significant increase in odds of asthma.

We found a significant association between 25(OH)D levels and odds of asthma in Pampas. These results replicate those found in a previous population-based study conducted in this community, where we found higher odds of asthma with lower 25(OH)D levels [7]. In the previous study, the prevalence of deficiency was similar to the prevalence in Pampas in the current study (47% vs. 52.7% in previous vs. current study) [7]. Furthermore, like the current study, the relationship between 25(OH)D levels and asthma was stronger among children with atopy [7]. Thus, our results confirm previous observations in Pampas and suggest that the relationship between 25(OH)D serum levels and asthma may be most relevant among children with allergic asthma. The sample size for children without atopy was smaller than those with atopy, which could explain the lack of statistical significance in this analysis. Future studies should consider stratification by atopy to capture differences among individuals with the allergic phenotype.

In Villa, we did not observe a significant relationship between asthma and 25(OH)D concentrations. Given that the prevalence of vitamin D deficiency was far lower in Villa, our results may indicate that vitamin D deficiency and prevalent asthma are more strongly associated in individuals with more severe deficiency. The populations of Pampas and Villa, while geographically adjacent, differ in several factors that may help explain the differing associations between communities and the different prevalences of deficiency. Villa is more urbanized than Pampas, with more major roadways and commercial activity and areas with higher population density. Furthermore, our data indicate that the population of Villa has higher overall SES and differing age structures, with the percentage of individuals over age 65 being over two times higher in Villa (~7%) vs. Pampas (~3%). Although we adjusted for SES, there may be lifestyle factors related to urbanization that influence vitamin D status not accounted for in our models. Nutritional factors such as magnesium and calcium intake have been hypothesized to influence vitamin D absorption. Data from a food frequency questionnaire in a subset of 646 children in our study show that in Villa, children consumed a higher mean daily frequency of vitamin D-rich oily fish and fortified milk in the 2 weeks prior to blood draw (Table 2), indicating that nutritional factors were likely important drivers of the differences in 25(OH)D distributions between communities. Physical activity levels also may differ between the two communities, leading to different levels of sun exposure, which may help explain the difference in the prevalence of deficiency. A higher proportion of the population of Pampas is composed of primarily highland migrants, with 81% of participants in Pampas vs. 48% in Villa (*p* < 0.001) reporting one or both parents being born outside of Lima. This difference indicates that a much higher proportion of children in Pampas were born of first generation rural-to-urban migrant parents. Thus, there may be genetic differences between the two populations that could modulate the relationship between vitamin D deficiency and asthma. Indeed, polymorphisms in several genes related to vitamin D’s function in the body can impact vitamin D status [17, 18], and there is evidence that variants in genes related to vitamin D status are associated with ancestry [19, 20].

Our findings suggest a U-or L-shaped ecological relationship between vitamin D deficiency and asthma prevalence, and a possible threshold effect for the protective benefit of vitamin D above concentrations occurring at approximately 27.5 ng/ml. Other studies have observed similar relationships between 25(OH)D levels and asthma outcomes. A study of 264 preschool children demonstrated a threshold effect for 25(OH)D levels and asthma exacerbations at 20 ng/ml [21]. Other studies have demonstrated a U-shaped relationship of 25(OH)D levels with allergic sensitization [22], serum IgE levels [23], and pulmonary function [24, 25]. There is a lack of consensus regarding optimal levels of circulating 25(OH)D concentrations for health outcomes related to the non-classical functions of vitamin D. However, the threshold observed in this study should be considered exploratory. The distributions of 25(OH)D levels between the two communities have little overlap; thus, the different relationship under and above the threshold may in fact be driven by underlying differences between the two community populations, such as genetic or lifestyle differences. Furthermore, in Pampas, blood draws for children with asthma and controls were not conducted in parallel, and we were thus unable to adjust by month, which could have resulted in residual confounding. However, all blood draws in Pampas were conducted during the same season. In Villa, blood draws for cases and controls were conducted in parallel.

We did not find evidence for an association between 25(OH)D concentrations and ACT score or measures of pulmonary function. Our results differ from a study by Gupta et al. [26], which found a positive relationship of 25(OH)D serum concentrations with pulmonary function and ACT score among children with asthma. Cremers et al. [27] found no association between maternal vitamin D intake and lung function in offspring at age 6–7 years. However, in a study of 1213 Canadian children, Niruban (2014) found a U-shaped relationship between children’s 25(OH)D levels and measures of pulmonary function (FEV_1_ and FVC), with children in low and high vitamin D categories having a reduced rate of change in lung function [25].

Our results also do not provide evidence for a relationship between 25(OH)D levels and FeNO. Fractional exhaled nitric oxide (FeNO) is a marker of lung inflammation and is known to be elevated in individuals with asthma [28]. Our results regarding FeNO are consistent with a study conducted by our group showing no association between 25(OH)D levels and FeNO [7, 29–31]. Our results also do not support an association between 25(OH)D concentrations and total serum IgE. The literature regarding vitamin D deficiency and markers of allergy has been mixed [6]. In a cohort of 616 Costa Rican children, total serum IgE and 25(OH)D were inversely associated [32]. Conversely, one study of 7288 British adults demonstrated a U-shaped relationship between 25(OH)D and total serum IgE; elevated serum IgE was associated with both low and high 25(OH)D concentrations [23]. In a study conducted by our group in Peru, we did not observe an association between IgE and total 25(OH)D levels [7], consistent with current findings. Well-designed, prospective studies and supplementation trials are needed to clarify the relationship between vitamin D deficiency and allergy.

There are limitations to this study. We did not adjust for genetic factors associated with vitamin D deficiency and asthma risk, and lifestyle factors, such as general dietary factors, sun exposure, and physical activity levels are not accounted for in our analyses. Of note, the study population had negligible reported use (<5%) of inhaled corticosteroids, virtually eliminating corticosteroid use as a confounder. In addition, a higher percentage of children without asthma had their blood drawn during the fall season as compared to summer, which could have led to a conservative bias and may help explain our observing no association between asthma and 25(OH)D concentrations in Villa El Salvador. Our definition of current asthma, based on the presence of wheeze or medication use in the prior 12 months, could have led to the inclusion of non-asthma cases in the asthma group. However, 92.3% of children with current asthma also had a previous physician diagnosis, indicating that misclassification was likely minimal in our study population. Another limitation of this study is the lack of information regarding sun exposure levels, which could have acted as confounding factor in our analyses.

An important limitation of cross-sectional studies is the inability to assess temporality or causality. For this reason, well-designed, large-scale randomized controlled supplementation trials are needed to clarify the relationship between vitamin D deficiency and asthma and allergy, particularly the effect of maternal supplementation on disease risk in offspring. Studies examining exposures beyond 4 years of age may be missing the critical window of exposure for determining risk in later life, since the prenatal period and the first 1000 days of life are hypothesized to be etiologically relevant for determining asthma and allergy risk [33]. There is, however, significant evidence that vitamin D supplementation can improve asthma control and reduce of exacerbations. A recent Cochrane systematic review and meta-analysis showed with high quality evidence that vitamin D supplementation significantly reduced the risk of asthma exacerbations requiring oral corticosteroids (rate ratio 0.63, 95% CI: 0.45 to 0.88) [34]. The risk of hospitalization was also significantly reduced, although the evidence was of moderate quality [34]. Thus, vitamin D supplementation has the potential to play a role in asthma management, which, given the relative affordability and safety of vitamin D supplements, could have profound implications in low-resource settings.

This study enrolled a large sample of children. Furthermore, this study contributes important information regarding the prevalence of vitamin D deficiency in Latin American countries, which is decidedly lacking. Given that the selection criteria were minimal for our study and that we observed a wide range of 25(OH)D values, we believe our results to be generalizable to other pediatric populations, barring any relevant genetic differences.

## Conclusion

The results of our study suggest that the relationship between 25(OH)D levels and asthma may be stronger among populations with a higher prevalence of deficiency, and among individuals with the allergic phenotype. Combined data from both study sites suggests that the relationship between 25(OH)D levels and asthma displays a threshold effect at approximately 27.5 ng/ml. Our study does not support associations of total serum 25(OH)D concentrations with pulmonary function measures, asthma control, or markers of lung inflammation (FeNO) or allergy (IgE). There is a need for comprehensive birth cohort studies examining exposures in utero and early life, including nutritional factors such as vitamin D deficiency. Randomized controlled trials conducted in children to determine the effect of vitamin D supplementation on incident asthma and asthma severity and control are also needed.

## Additional file

Additional file 1: Table S1. Online Supplement: Results of sensitivity analyses; Description of data: Results of sensitivity analyses for excluding children with previous asthma from main analyses. (PDF 92 kb)

## Abbreviations

ACT: Asthma Control Test
BMI: Body Mass Index
FeNO: Fractional exhaled nitric oxide
FEV_1_: Forced expiratory volume in 1 second
FVC: Forced vital capacity
IgE: Immunoglobulin E
SES: Socioeconomic status
