# Supplementary material for: Associations between serum 25(OH)D concentrations and prevalent asthma among children living in communities with differing levels of urbanization: a cross-sectional study
Source: Asthma Res Pract. 2017 Jun 2;3:5. doi: 10.1186/s40733-017-0033-2 (PMC5457560; doi:10.1186/s40733-017-0033-2)
Supplement: Additional file 1: Table S1. — Online Supplement: Results of sensitivity analyses; Description of data: Results of sensitivity analyses for excluding children with previous asthma from main analyses. (PDF 92 kb) [file 40733_2017_33_MOESM1_ESM.pdf]

ONLINE SUPPLEMENT

e-Table 1. Sensitivity analysis of excluding previous asthma cases in multivariable logistic regression analysis of the association between 25-OH vitamin D levels and asthma.

	**Adjusted* relationships with total serum 25(OH)D levels (per 10 ng/ml decrease)**					
	**Overall (n=884)**		**Atopy (n=603)**		**No Atopy (n=262)**	
	**Pampas (n=419)**	**Villa (n=465)**	**Pampas (n=293)**	**Villa (n=310)**	**Pampas (n=118)**	**Villa (n=144)**
**Asthma**	1.18 (1.02 to 1.38)<br>0.03	0.954 (0.867 to 1.05)<br>0.33	1.18 (0.985 to 1.42)<br>0.07	0.925 (0.829 to 1.03)<br>0.16	1.07 (0.806 to 1.43)<br>0.63	1.06 (0.86 to 1.32)<br>0.59
	**(n=354)**	**(n=385)**	**(n=256)**	**(n=257)**	**(n=98)**	**(n=128)**
**Asthma (excluding previous cases)**	1.45 (1.00 to 2.10)<br>0.05	0.857 (0.667 to 1.10)<br>0.23	1.34 (0.88 to 2.06)<br>0.17	0.799 (0.596 to 1.07)<br>0.13	1.37 (0.685 to 2.73)<br>0.38	1.00 (0.561 to 1.79)<br>0.99
